# Occurrence of Patulin and Polyphenol Profile of Croatian Traditional and Conventional Apple Cultivars during Storage

**DOI:** 10.3390/foods11131912

**Published:** 2022-06-27

**Authors:** Ana-Marija Gotal Skoko, Ružica Vilić, Marija Kovač, Ante Nevistić, Bojan Šarkanj, Marta Lores, Maria Celeiro, Martina Skendrović Babojelić, Tihomir Kovač, Ante Lončarić

**Affiliations:** 1 Faculty of Food Technology Osijek, Josip Juraj Strossmayer University of Osijek, Franje Kuhača 18, 31000 Osijek, Croatia; amgotal@ptfos.hr (A.-M.G.S.); ruzica.vilic@ptfos.hr (R.V.); ante.loncaric@ptfos.hr (A.L.); 2 Department of Food Technology, University Centre Koprivnica, University North, Trg dr. Žarka Dolinara 1, 48000 Koprivnica, Croatia; marija.kovac@unin.hr (M.K.); bsarkanj@unin.hr (B.Š.); 3 Inspecto Ltd., Industrijska Zona Nemetin, Vukovarska Cesta 239b, 31000 Osijek, Croatia; ante.nevistic@inspecto.hr; 4 LIDSA-CRETUS, Department of Analytical Chemistry, Universidade de Santiago de Compostela, E-15782 Santiago de Compostela, Spain; marta.lores@usc.es (M.L.); maria.celeiro.montero@usc.es (M.C.); 5 Department of Pomology, Faculty of Agriculture, University of Zagreb, Svetošimunska 25, 10000 Zagreb, Croatia; mskendrovic@agr.hr

**Keywords:** phenolics, Croatian traditional apple cultivars, HPLC, patulin

## Abstract

Apples and apple-based products are among the most consumed fruits around the world. However, they are susceptible to infection with the fungi *Penicilium expansum*. In addition to the reduction of apple quality, secondary metabolism of this fungus produces a mycotoxin patulin that has a negative effect on human health. Currently, there is no available research in the literature on the resistance of Croatian traditional apple cultivars to contamination with *P. expansum*, and consequently, on the patulin content in apples and apple juice produced from those apples. Although the mechanism of apple resistance to fungal diseases has not yet been sufficiently investigated, some studies have shown that polyphenolic compounds have some impact on fungi growth. In order to contribute with new knowledge, this research deals with monitoring the growth of *P. expansum* on apples, patulin detection by LC/MS-MS, determination of polyphenol profile by validated HPLC method, and determining the effect of polyphenolic compounds on fungi growth and patulin production during apple storage. The results of this study have shown that Croatian traditional apple cultivars harvested from family farm Horvatić contain higher concentration of polyphenolic compounds and higher antioxidant activity. At the same time, they showed more resistance to infection by *P. expansum* than conventional ones. The higher content of dihydrochalcones and flavanols encouraged the biosynthesis of patulin in examined cultivars. However, the higher content of non-flavonoids such as 2-6 dimethoxybenzoic acid, 4-hydroxycinnamic acid and chlorogenic acid leads to decrease in content of patulin. In conclusion, it seems that content of polyphenols and patulin production are correlated.

## 1. Introduction

Apples are among the most consumed fruit worldwide with significant source of varied biologically active compounds, such as polyphenols [1]. Fresh fruit consumption represents the main source of polyphenols from apple throughout the year. Polyphenols are among the most ubiquitous groups of plant secondary metabolites found in natural food sources [2]. Apple polyphenols can participate in a series of reactions such as quenching singlet oxygen and acting as chelators and trapping free radicals, or as reducing agents by donating hydrogen [3]. Polyphenols also can promote human health due to their antioxidant, antiviral, and anti-inflammatory properties, among other health beneficial effects [4]. Traditional and conventional apple cultivars have the same groups of polyphenols, being mainly phenolic acids, flavonols, dihydrochalcones, flavan-3-ols and anthocyanins. Despite the similarities, traditional apple cultivars proved to be rich in some individual polyphenolics, such as epicatechin, quercetin-3-rutinoside, chlorogenic acid, procyanidins B1, B2, A2, etc. [5,6]. The content of phenolic acids and flavanols of conventional apple cultivars were reduced by breeding, which lead to a decrease in enzymatic browning and astringent taste [7,8]. However other important factors influencing the content of polyphenols in the apple are geographic location, weather conditions of the harvesting season, agricultural conditions, fruit maturity, rootstocks, cultivar properties, crop load, development of infection, fruit position within the canopy. For example, it was found that a fluctuating temperature resulted in better colour and higher anthocyanin concentrations for apple fruits harvested from different areas [9]. Traditional apple cultivars are locally adapted to its natural environment they are mostly related to specific region, grown in backyards and small orchards. Polyphenols are induced in plants by various biotic and abiotic stresses and cold treatments or drought stress cause increases in levels of (-)-epicatechin and quercetin-3-galactoside in *Crataegus laevigata* and *C. monogyna*. These types of treatments also enhance the antioxidant capacity of the shoot extracts and may be the primary function of these cold-inducible flavonoids. Furthermore, investigation conducted by Mihaljevic et al. [10] showed that traditional apple cultivar are more resistant to drought when compared with commercial one.

The shelf life of apples can be increased by storing them under certain conditions, such as controlled, modified and dynamic controlled atmosphere, initial low oxygen stress, ultra-low oxygen etc. [11,12]. Furthermore, apples are highly vulnerable to storage disorders during long-term storage. To avoid postharvest losses, they are kept in cold storage at around 0 °C for several months [9]. Unfortunately, long-term storage can also cause side effects such as watercore, superficial scald and internal browning [13]. Quality of apples changes quickly during storage and it cannot be improved, but it can somewise maintained during storage [1]. The polyphenol stability is decisive for the nutritional value of foods. However, they are considered unstable and highly prone to degradation or reaction with some factors, such as oxygen during processing and storage and/or metal ions, resulting in change in their structures. Therefore, the stability of polyphenols under various conditions is one of the most significant aspects, which includes temperature, light, pH, enzymes, metal ions, oxygen, proteins, or interaction with other food constituents [2]. During the storage, temperature has a great influence on polyphenols. Therefore, the strict temperature control is important to sustain the levels and stability of polyphenols. Radenkovs and Juhnevica- Radenkova [14] and Galani et al. [15] reported that total polyphenols increase during 15 days storage at 4 °C. They concluded that apples during storage were affected by storage technology and harvesting time [14]. Addie et al. [16] reported that cold storage did not have a significant effect on the 2′-O-glucose (phloridzin), cyanidin galactoside and total quercetin glycoside in the examined apple cultivars, corresponding to what was observed during controlled atmosphere storage. On the other hand, the increase in polyphenol oxidase (PPO) and peroxidase (POD) activity during storage can result in the degradation of polyphenols such as anthocyanins which leads to discoloration and loss of antioxidant activity. This is due to the ability of PPO and POD to destroy covalent bonds between anthocyanin glycosides, leading to sugar residues and aglycone [17]. Furthermore, the stability and antioxidant activity of polyphenols during storage can also be changed by chemical modifications, such as acylation, pigmentation, glycosylation and hydroxylation. These modifications increase the stability of polyphenols, except hydroxylation which leads to a reduction of the stability [18]. As an example, acylated anthocyanins showed higher stability than their corresponding anthocyanins [19]. Other studies have shown that amino acids, flavonols, cinnamic acid and benzoic acid also react with anthocyanins due to the acylation of organic acids [20]. Furthermore, flavonoids are known to protect oxidation of vitamins C and E during apple storage [21]. Łysiak et al. [22] reported that after storage the content of chlorogenic acid and phloridzin decreased in all groups of apples, except phloretin 2′-O-xyloglucose, which increased by 20%. The biggest degradation of total dihydrochalcones was observed in apples with the shortest period of growth. Except all storage conditions that are mentioned above, the quality of apple is also affected by harvesting conditions which lead to unintended consequences during storage. About 20–30% of fresh fruits are lost after harvest due to the decay induced by fungal pathogens [23]. Despite the many types of pathogens that cause postharvest decay of fruit, *Penicillium expansum* is one of the most damaging postharvest pathogens. *P. expansum* is the main producer of the dominant mycotoxin patulin that contaminates fruits and fruit-derived products, especially apples [24,25]. Traditional apple varieties contain higher amount of polyphenolic compounds, which contribute to the resistance of apple to plant diseases. Due to higher levels of polyphenols, flavan-3-ols, flavanols, procyanidins, phenolic acids and dihydrochalcones, traditional apple varieties showed great resistance for *P. expansum* infection [25]. Our previous studies showed that higher content of polyphenols, specially flavan-3-ols, induce patulin production. Furthermore, the biosynthesized patulin concentration in examined apple cultivars were boosted with higher content of catechin, gallic acid and epicatechin. In conclusion, that is confirmation of pro-oxidant activity of polyphenolic compounds and susceptibility of *Penicillium expansum* cells to the disturbance of oxidative status [26].

The purpose of the present study is to broaden the knowledge of the resistance of traditional apples from Croatia by determining the effect of cold storage on polyphenol profile and occurrence of mycotoxin patulin in traditional and conventional apple cultivars from Croatia.

## 2. Materials and Methods

### 2.1. Chemicals

Folin−Ciocalteu reagent was purchased from Kemika (Zagreb, Croatia). Methanol (MS grade), employed as extraction solvent was supplied by Scharlaub (Chemie S.A., Barcelona, Spain), ultrapure water was produced in the laboratory with a Milli-Q gradient system (Millipore, Bedford, MA, USA) and the formic acid was supplied by Merck (Darmstadt, Germany). Catechin (CAS: 154–23–4, ≥99.0%), epicatechin (CAS: 490–46–0; ≥98%), phloretin (CAS: 60–82–2, ≥99.0%), phloridzin (CAS: 60–81–1, ≥98%), procyanidin B1 (CAS: 20315-25-7, ≥90%), procyanidin B2 (CAS: 29106-49-8, ≥90%), procyanidin A2 (CAS: 41743-41-3, ≥99.0%), quercetine (CAS: 117-39-5, ≥95%), quercetin 3-β-D-glucoside (CAS: 482-35-9, ≥90%), quercetin-3-rutinoside (CAS: 207671-50-9, ≥94%), myricetin (CAS: 529-44-2, ≥96.0%), chlorogenic acid (CAS: 327–97–9, ≥95%), 4-hydroxycinnamic acid (CAS: 7400-08-0, ≥98%), 2-6-dimethoxybenzoic acid (CAS: 1466-76-8, ≥98%) and patulin (CAS: 149-29-1, ≥98.0%) were obtained from Sigma–Aldrich (Chemie Check GmbH, Steinheim, Germany).

### 2.2. Plant Material

Conventional apple cultivars, ‘Idared’, ‘Jonagold’, ‘Golden Delicious’, ‘Granny Smith’ and ‘Fuji’ were purchased in September last year from a local market in the maturity stage from OPG (family farm) Pavičić, Petrijevci, Osijek, Croatia. The traditional apple cultivars, ‘Petrovnjača’, ‘Adamovka’, ‘Amovka’, and ‘Srčika’ and ‘Wagner’ were collected in September from OPG Horvatić, Cvetkovac, 48,312 Rasinja (46°20′90.3″ N 16°69′95.9″ E), Croatia. The orchard is located in an area of temperate continental climate, and the soil type is pseudogley, clayey-loamy texture, slightly acidic to neutral reaction. Cultivars were grafted on wild apple rootstocks and trees were planted at distance about 6 m between rows and 5 m within rows. All studied apple cultivars were authenticated by a pomologist. Traditional and conventional apple cultivars were stored in a refrigerator on 4 °C during 3 and 6 months. Extraction of bioactive polyphenolic compounds was performed by Ultrasound. Average sample was prepared from 10 apples of each cultivar which were previously lyophilized (Christ, Osterode am Harz, Germany) and pulverised. The 250 mg of average sample was mixed with extraction solvent (80% MeOH in water). Ultrasound-assisted extraction (UAE) was performed in an ultrasonic bath at 35 kHz for 15 min in 20 mL test tubes. The procedure can be found in our previous work Lončarić et al. [26]. Each extraction was performed in triplicate.

### 2.3. Total Polyphenol Determination

The total polyphenol content in the traditional and conventional apple were measured by using Folin-Ciocalteu method with several modifications. The procedure can be found in our previous work Lončarić et al. [27]. The absorbance was read at 765 nm by spectrophotometer (Jenway 6300, Bibby Scientific, Stone, UK). For each sample, the measurements were performed in triplicates and the average value was interpolated on a gallic acid calibration curve and expressed as mg of gallic acid equivalents per L of extract (mg/L).

### 2.4. Identification of Polyphenols by LC-DAD Analysis

Polyphenol identification was performed on a Jasco LC Net II, equipped with the AS-4150 autosampler, the PU-4180 pump and the MD-4010 PDA detector. The method and instrumental conditions were previously optimised (A1-A5) by the authors Lončarić et al. [27]. Quantification has been performed by external standard calibration and the calibration range for each phenolic standard was 0.1–10 µg/g. The concentration of individuals polyphenols was expressed as µg/g of dry weight (dw).

### 2.5. Patulin Determination by UHPLC-MS/MS Analysis

Confirmatory UHPLC-MS/MS analytical method was used for patulin determination, successfully in-house validated and fitted to purpose. A Waters Acquity H-class UPLC system (Waters, Milford, MA, USA) was employed to perform the chromatographic separation using a BEH C18 column (100 × 2.1 mm, 1.7 µm particle size) (Waters, Milford, MA, USA) maintained at 40 °C. The UPLC system was coupled to Waters Xevo TQD tandem mass spectrometer (Waters, Milford, MA, USA) equipped with an electrospray ionization (ESI) interface operating in negative ionization mode. The procedure and description of the method can be found in our previous work Lončarić et al. [27]. Quantification was performed by external solvent calibration curve covering a patulin concentration range between 10 and 100 µg/kg, while applying a dilution step for samples with concentrations outside this specified range. The concentration of patulin was expressed as µg/g of dw.

### 2.6. Statistical Analysis

Data presented in this work are expressed as the mean value ± SEM (standard error of measurement) from three separate experiments. The pooled datasets were checked for distribution normality by Shapiro-Wilk test and compared by nonparametric statistics methods. Differences between traditional and conventional varieties were compared by Mann-Whitney U test, and the box plot diagrams were drawn. Statistical analyses were performed using the Statistica 13.5 (TIBCO Software Inc., Palo Alto, CA, USA) and differences were considered statistically significant when the *p* value was < 0.05. Correlogram was prepared by using Minitab Statistical Software (Minitab LLC, State College, PA, USA).

## 3. Results and Discussion

**Figure 1:**
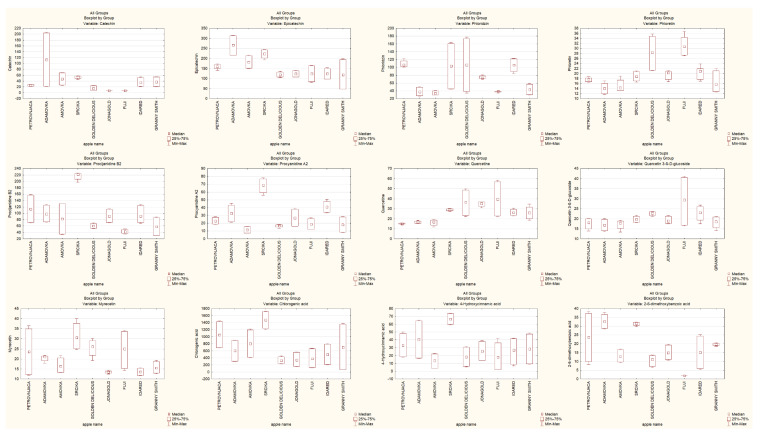
The content and ratio of identified polyphenols and patulin in traditional and conventional apple cultivars.

In this study we investigated the content of total polyphenols, flavanols, dihydrochalcones, procyanidins, flavonols, non- flavonoids and the content of patulin in examined Croatian traditional and conventional apple cultivars after harvest, and after three and six months of storage. The content and ratio of identified polyphenols and patulin in traditional and conventional apple cultivars is given in Figure 1. The highest content of total polyphenols in traditional apple cultivars was detected after three months of storage, corresponding to ’Srčika’ (81.39 ± 1.18 mg/100 g DW followed by ’Adamovka’ (61.30 ± 3.63 mg/100 g DW). In other traditional apple cultivars, the content of total polyphenols was the highest after harvest and it decreased during storage. Despite that, traditional apple cultivars had higher content of polyphenols during storage than conventional ones. In the conventional apple cultivars, the highest content of polyphenols had ’Idared’ (43.70 ± 3.35 mg/100 g DW) after harvest, followed by ’Granny Smith’ (42.03 ± 2.81 mg/100 g DW) after three months of storage (Table 1). These results are in accordance with the content of total polyphenols reported by Akagić et al. [28].

Furthermore, the content of the most abundant polyphenols found in apples, catechin and epicatechin, were determined. The content of catechin and epicatechin in both traditional and conventional apple cultivars decreases during storage (Table 2). The highest content of catechin and epicatechin in traditional apple cultivars was detected in ’Adamovka’ (204.07 and 313.54 µg/g dw, respectively) followed by lower content of catechin in ’Amovka’ (67.55 µg/g dw) and lower content of epicatechin in ’Srčika’ (242.53 µg/g dw). On the other hand, the highest content of catechin in conventional apple cultivars was detected in ’Granny Smith’ (53.69 µg/g dw) followed by ’Idared’ (51.38 µg/g dw). Furthermore, there were some apple cultivars such as ’Jonagold’ and ’Fuji’ where catechin was not detected. The highest content of epicatechin was detected in ’Granny Smith’ (198.44 µg/g dw) after three months of storage. In general, traditional apple cultivars are richer in catechins and epicatechins than conventional ones. These results compliance with the content of catechin and epicatechin reported in our previous study [26]. Where the content of epicatechin in ’Granny Smith’ was 201.72 ± 5.61 µg/g dw and the catechin in ’Jonagold’ and ’Fuji’ was not also detected. The concentration of epicatechin in all traditional apple cultivars were higher than the content of catechin in the same apples. For example, the content of catechin and epicatechin for ’Apistar’ was 512.36 ± 12.20 µg/g dw for catechin and 1194.72 ± 21.41 µg/g dw for epicatechin.

Regarding dihydrochalcones, phloretin and phloridzin were detected. Phloridzin can cause low sensitivity of apple cultivars to the most important diseases and provides resistance to the common apple pathogens [28]. In both, traditional and conventional apple cultivars, there is a decrease in phloridzin and phloretin during storage (Table 3). The highest content of phloridzin in traditional apple cultivars was detected in ’Srčika’ (161.37 µg/g dw) after harvest, followed by ’Petrovnjača’ (116.64 µg/g dw) after six months of storage. The highest content of phloretin had ’Adamovka’ (35.8 µg/g dw) after three months of storage, followed by ’Srčika’ (20.75 ± 0.25 µg/g dw) after harvest. In conventional apple cultivars the highest content of phloridzin and phloretin was detected in ’Golden Delicious’ (173.99 µg/g dw; 34.94 µg/g dw) [29]. These results are in accordance with previously reported results of Lončarić et al. [27], Preti et al. [30] and Jakobek et al. [31].

Procyanidins B1, B2 and A2, were also detected in traditional and conventional apple cultivars (Table 4). Procyanidins are the major class of apple polyphenols, and they present a good substrate for polyphenol oxidase which leads to their involvement in enzymatic oxidation [7,32,33]. In both, traditional and conventional apple cultivars, there is a decrease in the content of procyanidins B1, B2 and A2 during storage. Łysiak et al. [22] also reported that the content of procyanidins in traditional and conventional cultivars decreased during storage. The highest content of procyanidins B1, B2 and A2 in traditional apple cultivar was detected in ’Srčika’ (244.51; 221.83; 77.07 µg/g dw, respectively). As for conventional apple cultivars, the highest content of procyanidins B1, B2 and A2 was detected in ’Idared’ (184.69; 121.72; 48.49 µg/g dw, respectively). Traditional apple cultivars had a significantly higher content of procyanidins than conventional ones. Such correlation was also reported by Jakobek et al. [34]. Flavonols were also detected in traditional and conventional apple cultivars, specifically quercetine, quercetin-3-ß-D-glucoside, quercetin-3-rutinoside and myricetin (Table 5). In traditional apple cultivars, the highest content of quercetine was detected in ’Srčika’ (29.9 µg/g dw), of quercetin-3- ß-D-glucoside in ’Adamovka’ (25.17 µg/g dw) and of quercetin-3-rutinoside and myricetin in ’Srčika’ (34.4 µg/g dw; 37.72 µg/g dw). On the other hand, the highest content of quercetine, quercetin-3- ß-D- glucoside, quercetin-3-rutinoside and myricetin in conventional apple cultivars was detected in ’Fuji’ (57.18; 40.63; 59.54; 33.75 µg/g dw, respectively). In some apple cultivars there is an increase in flavonols during the first three months of storage, but by the end of the sixth month of storage their content decreases significantly. Łysiak et al. [22] reported that the content of flavonols significantly decreased after storage, being almost 1.6 times lower than before storage. The non-flavonoids chlorogenic acid, 4-hydroxycinnamic acid and 2-6-dimethoxybenzoic acid were also detected in traditional and conventional apple cultivars (Table 6). The highest content of chlorogenic and 4-hydroxycinnamic acid in traditional apple cultivar was detected in ’Srčika’ (1715.54 and 73.56 µg/g dw, respectively). Moreover, the highest content of 2-6-dimethoxybenzoic acid had ’Petrovnjača’ (37.38 µg/g dw). In conventional apple cultivars the highest content of chlorogenic and 4-hydroxycinnamic acid was detected in ’Granny Smith’ (1356.68 and 47.79 µg/g dw, respectively) and considering 2-6-dimethoxybenzoic acid, the cultivar ’Idared’ had the highest amount 24.45 µg/g dw. On the other hand, there was a conventional cultivar ’Fuji’ in which no 2-6-dimethoxybenzoic acid was detected. Obtained results show that traditional apple cultivars have a higher content of non- flavonoid than conventional ones [7,30,35,36]. Other authors also observed that traditional apple cultivars are dominated by non-flavonoids and conventional apple cultivars are dominated by flavanols in different apple cultivars [37,38,39].

The content of mycotoxin patulin in traditional and conventional apple cultivars was also measured. The extent of host contamination by *P. expansum* is the result of the interaction between fruit resistance and pathogen virulence. The patulin content was determined at the end of incubation period, after harvest and after six months of storage. The resistance of traditional and conventional apple cultivars to infection by *P. expansum* CBS 325.48 was performed using 168 h old *P. expansum* grown on potato dextrose agar in Petri’s dish at 29 °C. Furthermore, inoculated 1 cm thick apple slices were incubated at 29 °C until the *P. expansum* colony reaches the diameter of 9 cm. The content of patulin was determined by UPLC-MS/MS method. The results of patulin content are presented in Table 7. The highest content after harvest was measured in conventional apple cultivars in ’Fuji’ (289.2 µg/g dw), followed by ’Granny Smith’ (180.35 µg/g dw). In traditional apple cultivars the highest content was observed in ’Petrovnjača’ (23.50 µg/g dw). After six month of storage the highest content and above the regulated level of 25 µg/kg for solid apple products was measured in conventional apple cultivars [40]. The highest content of patulin in conventional apple cultivars was measured in ’Fuji’ (3425.75 µg/g dw), followed by ’Idared’ (2750.90 µg/g dw). In traditional apple cultivars the content of patulin was much lower, although above the regulated level. The highest content of patulin was detected in ’Petrovnjača’ (238.10 µg/g dw). In the samples of traditional apple cultivar ’Adamovka’ patulin was not detected after six months of storage. It should be highlighted the fact that Adamovka does not contain patulin at the start and does not become infected during 6 months of storage. Furthermore, after 6 month of storage the patulin content in traditional apple cultivars vs. conventional are strikingly lower (with the exception of ‘Jonagold’ and, to a lesser extent, ‘Golden Delicious’).

**Figure 2:**
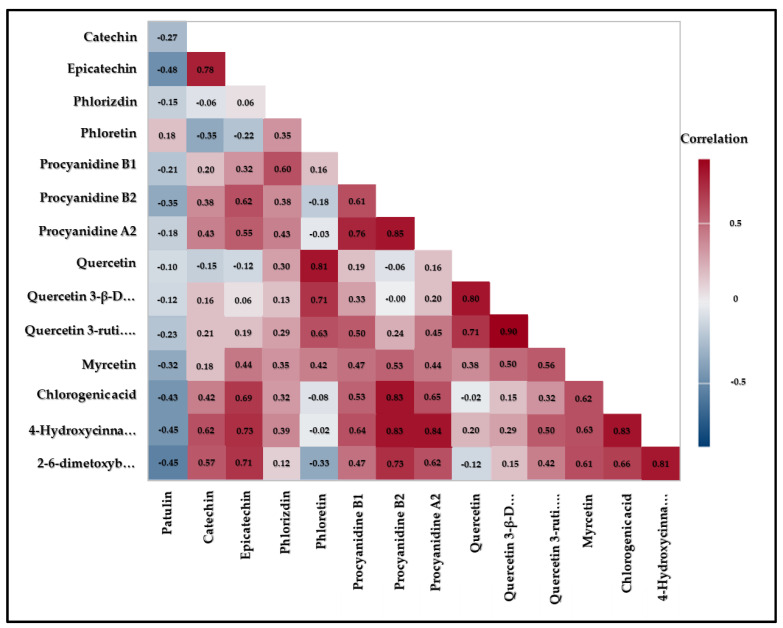
The colour map of correlations of patulin with identified polyphenols.

The interplay of pro-oxidant activity of polyphenolic compounds and sensitivity of *P. expansum* cells to the disturbance of oxidative status is expected to affect the patulin biosynthesis. The correlation between individual polyphenol content and patulin detected concentrations are presented in Figure 2.

The highest content of patulin was negatively correlated with higher content of non-flavonoids such as 2-6-dimethoxybenzoic acid (r = −0.5566, *p* = 0), 4-hydroxycinnamic acid (r = −0.4485, *p* = 0) and chlorogenic acid (r = −0.4424, *p* = 0) (Table A1). This means that increasing the content of non-flavonoids leads to decrease in content of patulin. This is in accordance with the above commented fact that traditional apple cultivars had a higher content of non-flavonoids and therefore a lower content of patulin than conventional ones. The highest content of patulin was positively correlated with higher content of flavanols and dihydrochalcones. Such correlation of results originates from the pro-oxidative effect of flavanols that induce the accumulation of reactive oxygen species in *P. expansum* cells, thus triggering the cellular antioxidant defence system and induced patulin biosynthesis, a secondary defence system that lowers reactive oxygen species levels in the cells [41,42]. Antioxidant defence systems can act as reactive oxygen species scavenges and they are closely combined with the pathogenicity of *P. expansum* [43]. Moreover, dihydrochalcones also showed a positive correlation, especially phloridzin, the major polyphenol responsible for apple resistance to fungal infection. Due to the formation of the hydrolysed product, phloretin, which then oxidizes and forms fungitoxic o-quinone [44,45].

## 4. Conclusions

The research conducted within this study provided the data on patulin occurrence and polyphenol profile of Croatian traditional and conventional apple cultivars during storage. It was shown that traditional apple cultivars contain higher concentration of polyphenolic compounds, and at the same time, they are more resistant to infection by *Penicillium expansum*. However, it seems that higher content of polyphenols induces patulin production. Moreover, the higher content of flavanols and dihydrochalcones boosted the biosynthesized patulin concentration in examined cultivars, probably due to their pro-oxidant activity and sensitivity of *P. expansum* cells to the disturbance of oxidative status. On the other hand, the higher content of non-flavonoids led to decrease in patulin content. This could be the confirmation that traditional apple cultivars are more resistant to *P. expansum* due to higher content of non-flavonoids. Future research will be performed to contribute the food and feed safety from the point of view of patulin biosynthesis, help preventing economic losses and contributing the global food sufficiency.

## Figures and Tables

**Table 1:** Total polyphenol content (mg/100 g DW) in the traditional and conventional apple cultivars.

	Cultivar	Month		
		0	3	6
**Traditional**	‘Petrovnjača’	50.34 ± 3.99	47.13 ± 2.72	41.59 ± 2.45
	‘Adamovka’	50.75 ± 3.72	61.30 ± 3.63	36.39 ± 2.63
	‘Amovka’	22.74 ± 3.72	29.95 ± 2.54	22.39 ± 1.27
	‘Srčika’	60.40 ± 0.73	81.39 ± 1.18	70.53 ± 4.35
**Conventional**	‘Golden Delicious’	32.52 ± 1.45	36.17 ± 2.9	28.64 ± 1.36
	‘Jonagold’	35.66 ± 2.9	35.46 ± 2.36	24.05 ± 1.45
	‘Fuji’	37.07 ± 2.36	23.73 ± 1.45	14.63 ± 1.27
	‘Idared’	43.70 ± 3.35	35.08 ± 0	30.78 ± 0.91
	‘Granny Smith’	37.64 ± 1.63	42.03 ± 2.81	26.68 ± 3.08

Mean ± SD based on two extracts each measured twice (*n* = 4).

**Table 2:** The content of flavanols (µg/g dw ^a^) in the traditional and conventional apple cultivars.

Compound		Catechin			Epicatechin		
Month		0	3	6	0	3	6
**Traditional**	‘Petrovnjača’	28.30 ± 0.26	22.94 ± 0.48	22.87 ± 0.59	170.61 ± 2.17	159.56 ± 3.02	148.07 ± 5.65
	‘Adamovka’	204.07 ± 2.51	33.36 ± 1.19	22.67 ± 0.73	313.54 ± 1.30	295.29 ± 6.62	217.69 ± 3.32
	‘Amovka’	67.55 ± 0.62	30.66 ± 0.58	26.47 ± 0.81	213.88 ± 2.59	172.70 ± 2.24	151.34 ± 0.41
	‘Srčika’	57.04 ± 0.45	51.89 ± 0.95	48.89 ± 2.43	242.53 ± 1.27	225.15 ± 4.23	200.76 ± 4.88
**Conventional**	‘Golden Delicious’	23.35 ± 0.80	n.d.	n.d.	133.16 ± 2.62	131.86 ± 5.38	107.22 ± 1.95
	‘Jonagold’	n.d.	n.d.	n.d.	139.00 ± 1.46	108.11 ± 1.05	107.93 ± 0.44
	‘Fuji’	n.d.	n.d.	n.d.	164.41 ± 1.37	103.68 ± 2.40	83.26 ± 2.13
	‘Idared’	51.38 ± 2.37	21.59 ± 0.87	22.48 ± 0.60	150.49 ± 2.14	107.85 ± 0.68	97.83 ± 0.35
	‘Granny Smith’	53.69 ± 0.73	41.96 ± 0.66	21.28 ± 0.64	194.68 ± 2.96	198.44 ± 4.89	n.d.

^a^: Dry weight. Mean ± SD based on two extracts each measured twice (*n* = 4). n.d.—not detected.

**Table 3:** The content of dihydrochalcones (µg/g dw ^a^) in the traditional and conventional apple cultivars.

Compound		Phloridzin			Phloretin		
Month		0	3	6	0	3	6
**Traditional**	‘Petrovnjača’	101.58 ± 1.25	79.76 ± 3.61	116.64 ± 5.45	18.22 ± 0.55	20.36 ± 0.70	16.97 ± 0.66
	‘Adamovka’	49.26 ± 1.07	77.27 ± 2.66	27.38 ± 0.31	11.93 ± 0.29	35.80 ± 0.42	16.26 ± 0.58
	‘Amovka’	39.60 ± 0.91	38.09 ± 3.33	29.67 ± 0.19	17.27 ± 1.87	16.88 ± 0.31	13.24 ± 0.07
	‘Srčika’	161.37 ± 1.67	101.43 ± 4.37	45.53 ± 1.54	20.75 ± 0.25	20.15 ± 0.19	16.96 ± 0.22
**Conventional**	‘Golden Delicious’	173.99 ± 2.88	136.13 ± 3.04	38.67 ± 3.83	34.94 ± 0.58	31.48 ± 1.65	21.49 ± 0.47
	‘Jonagold’	71.41 ± 0.19	75.01 ± 2.56	77.41 ± 4.23	20.79 ± 0.23	18.89 ± 1.02	18.00 ± 1.46
	‘Fuji’	39.28 ± 0.39	38.9 ± 4.44	36.19 ± 1.70	34.64 ± 1.70	30.08 ± 0.81	27.60 ± 0.67
	‘Idared’	121.72 ± 1.23	111.07 ± 3.42	88.49 ± 2.63	22.24 ± 1.20	22.70 ± 0.19	18.23 ± 1.62
	‘Granny Smith’	57.46 ± 1.06	29.56 ± 1.01	29.85 ± 0.41	20.66 ± 1.53	14.02 ± 0.57	12.78 ± 0.21

^a^: Dry weight. Mean ± SD based on two extracts each measured twice (*n* = 4).

**Table 4:** The content of procyanidins (µg/g dw ^a^) in the traditional and conventional apple cultivars.

Compound		Procyanidin B1			Procyanidin B2			Procyanidin A2		
Month		0	3	6	0	3	6	0	3	6
**Traditional**	‘Petrovnjača’	116.3 ± 2.98	128.92 ± 3.44	60.86 ± 0.61	156.11 ± 2.46	71.07 ± 1.32	71.48 ± 1.61	27.53 ± 0.75	27.63 ± 1.57	18.89 ± 0.50
	‘Adamovka’	84.76 ± 0.95	85.14 ± 0.82	57.02 ± 0.41	124.62 ± 2.40	109.20 ± 8.26	73.13 ± 1.61	43.53 ± 1.39	33.21 ± 1.61	21.98 ± 0.45
	‘Amovka’	50.68 ± 1.19	50.51 ± 0.36	50.98 ± 0.08	130.51 ± 0.34	77.29 ± 1.44	34.97 ± 0.92	15.6 ± 0.32	6.64 ± 0.03	7.37 ± 0.11
	‘Srčika’	244.51 ± 4.01	206.66 ± 5.30	60.60 ± 0.70	221.83 ± 4.61	216.36 ± 2.78	210.03 ± 14.25	77.07 ± 0.88	48.05 ± 1.42	58.92 ± 3.19
**Conventional**	‘Golden Delicious’	69.63 ± 0.46	67.04 ± 1.57	48.27 ± 0.41	67.62 ± 1.48	64.28 ± 18.81	52.96 ± 0.66	17.92 ± 0.46	17.02 ± 0.94	14.74 ± 0.93
	‘Jonagold’	129.87 ± 1.22	128.17 ± 0.73	52.63 ± 0.29	112.35 ± 1.13	77.58 ± 1.20	71.03 ± 0.55	38.1 ± 0.76	27.38 ± 1.27	16.30 ± 0.11
	‘Fuji’	97.59 ± 1.17	85.79 ± 2.38	60.74 ± 1.05	49.33 ± 1.30	45.42 ± 1.64	37.15 ± 1.17	25.96 ± 0.94	13.44 ± 0.40	12.09 ± 0.16
	‘Idared’	184.69 ± 4.00	122.96 ± 2.54	59.88 ± 0.50	121.72 ± 7.87	75.03 ± 2.49	70.91 ± 1.24	48.49 ± 1.6	35.65 ± 2.48	34.29 ± 0.71
	‘Granny Smith’	54.45 ± 0.37	55.74 ± 0.19	n.d.	86.86 ± 1.53	85.38 ± 1.10	30.78 ± 0.58	28.05 ± 0.9	26.68 ± 0.85	8.42 ± 0.37

^a^: Dry weight. Mean ± SD based on two extracts each measured twice (*n* = 4).

**Table 5:** The content of flavonols (µg/g dw ^a^) in the traditional and conventional apple cultivars.

Compound		Quercetine			Quercetin 3-β-D-Glucoside			Quercetin-3-Rutinoside			Myricetin		
Month		0	3	6	0	3	6	0	3	6	0	3	6
**Traditional**	‘Petrovnjača’	15.56 ± 0.35	16.43 ± 0.40	14.65 ± 0.28	19.93 ± 0.12	23.59 ± 0.30	15.05 ± 1.17	23.83 ± 0.57	32.09 ± 0.32	17.64 ± 1.65	35.25 ± 0.82	28.00 ± 0.93	12.26 ± 0.33
	‘Adamovka’	17.95 ± 0.40	20.75 ± 0.15	15.80 ± 0.42	19.57 ± 0.52	25.17 ± 0.96	14.13 ± 0.67	21.67 ± 1.66	26.16 ± 1.58	14.59 ± 0.16	21.47 ± 0.26	25.95 ± 1.90	19.35 ± 1.48
	‘Amovka’	18.53 ± 0.89	20.02 ± 0.15	13.74 ± 2.04	18.84 ± 0.36	17.40 ± 0.23	15.39 ± 2.25	18.11 ± 0.22	17.56 ± 0.34	16.43 ± 0.40	20.53 ± 1.29	18.20 ± 0.20	13.35 ± 0.10
	‘Srčika’	29.90 ± 0.34	26.80 ± 1.62	27.89 ± 0.32	21.26 ± 0.36	19.55 ± 1.01	18.26 ± 0.20	34.40 ± 0.95	30.60 ± 1.27	28.71 ± 1.89	37.72 ± 1.93	33.62 ± 1.46	25.26 ± 0.53
**Conventional**	‘Golden Delicious’	48.78 ± 0.66	40.99 ± 0.95	23.17 ± 1.13	23.54 ± 0.1	23.26 ± 0.23	21.72 ± 0.49	31.70 ± 0.86	27.79 ± 1.30	18.94 ± 0.32	29.01 ± 1.06	27.73 ± 1.58	21.82 ± 2.94
	‘Jonagold’	32.29 ± 1.03	40.75 ± 1.82	36.82 ± 0.14	21.08 ± 0.59	17.30 ± 0.43	17.61 ± 0.09	30.47 ± 1.63	12.95 ± 0.16	11.68 ± 0.04	13.92 ± 0.50	12.99 ± 2.22	12.93 ± 0.40
	‘Fuji’	57.18 ± 1.01	36.08 ± 1.43	22.86 ± 0.35	40.63 ± 0.32	36.97 ± 1.94	17.18 ± 0.85	59.54 ± 0.93	45.06 ± 1.91	15.81 ± 0.98	33.75 ± 0.33	31.24 ± 1.21	15.43 ± 1.27
	‘Idared’	29.45 ± 0.92	25.04 ± 0.96	23.44 ± 0.39	26.08 ± 0.67	25.12 ± 0.86	19.28 ± 1.49	41.86 ± 0.63	33.15 ± 1.47	18.32 ± 1.13	15.32 ± 0.24	16.02 ± 0.24	11.81 ± 0.20
	‘Granny Smith’	32.04 ± 1.93	23.00 ± 0.52	20.17 ± 1.71	20.67 ± 0.78	16.34 ± 0.37	15.69 ± 1.82	21.79 ± 1.20	18.27 ± 0.71	15.94 ± 1.11	18.64 ± 0.62	13.92 ± 0.27	12.97 ± 0.24

^a^: Dry weight. Mean ± SD based on two extracts each measured twice (*n* = 4).

**Table 6:** The content of non-flavonoids (µg/g dw ^a^) in the traditional and conventional apple cultivars.

Compound		Chlorogenic Acid			4-Hydroxycinnamic Acid			2-6-Dimethoxybenzoic Acid		
Month		0	3	6	0	3	6	0	3	6
**Traditional**	‘Petrovnjača’	1429.22 ± 16.03	1390.91 ± 12.48	690.21 ± 3.78	48.64 ± 1.01	46.60 ± 2.79	18.53 ± 0.37	37.38 ± 0.75	19.22 ± 1.32	9.73 ± 1.02
	‘Adamovka’	900.28 ± 7.24	421.94 ± 6.86	311.93 ± 6.60	64.58 ± 0.40	53.80 ± 1.02	16.75 ± 0.07	36.83 ± 0.72	31.12 ± 0.81	28.87 ± 0.42
	‘Amovka’	1194.39 ± 21.63	1085.78 ± 7.96	424.94 ± 10.69	22.29 ± 0.24	17.56 ± 0.87	3.86 ± 0.33	16.65 ± 0.51	16.56 ± 0.55	9.75 ± 0.29
	‘Srčika’	1715.54 ± 13.38	1651.14 ± 24.60	1236.17 ± 20.59	73.56 ± 0.76	67.98 ± 6.10	60.02 ± 0.56	32.15 ± 0.28	31.07 ± 0.95	30.60 ± 0.63
**Conventional**	‘Golden Delicious’	433.97 ± 18.74	427.05 ± 13.86	236.89 ± 6.48	30.68 ± 0.69	24.10 ± 0.58	6.32 ± 0.74	13.27 ± 0.41	8.62 ± 0.28	7.59 ± 1.16
	‘Jonagold’	556.36 ± 17.11	438.22 ± 16.07	159.39 ± 5.61	38.06 ± 0.95	24.78 ± 1.03	13.89 ± 0.03	19.28 ± 0.43	15.41 ± 1.04	11.13 ± 0.18
	‘Fuji’	661.76 ± 19.62	371.43 ± 13.57	123.21 ± 1.74	37.02 ± 3.74	13.34 ± 0.52	n.d.	n.d.	n.d.	n.d.
	‘Idared’	790.01 ± 11.04	695.96 ± 6.87	215.60 ± 7.05	41.77 ± 0.47	18.93 ± 0.97	9.52 ± 2.81	24.45 ± 0.60	7.12 ± 0.44	5.92 ± 0.42
	‘Granny Smith’	1356.68 ± 19.16	251.74 ± 4.45	65.01 ± 1.05	47.79 ± 0.69	46.63 ± 1.11	9.44 ± 0.54	19.12 ± 0.41	19.43 ± 0.18	20.21 ± 0.42

^a^: Dry weight. Mean ± SD based on two extracts each measured twice (*n* = 4). n.d.—not detected.

**Table 7:** The content of patulin (µg/kg dw ^a^) in the traditional and conventional apple cultivars.

	Cultivar	Month		
		0	3	6
**Traditional**	‘Petrovnjača’	23.5 ± 0.28 ^d^	n.d.	238.1 ± 5.52 ^f^
	‘Adamovka’	n.d.	n.d.	n.d.
	‘Amovka’	16.6 ± 0.28 ^de^	n.d.	28.7 ± 3.54 ^h^
	‘Srčika’	n.d.	n.d.	85.15 ± 0.21 ^g^
**Conventional**	‘Golden Delicious’	19.05 ± 0.21 ^d^	n.d.	294.05 ± 0.21 ^e^
	‘Jonagold’	120.5 ± 7.64 ^c^	n.d.	36.85 ± 1.06 ^gh^
	‘Fuji’	289.2 ± 19.66 ^a^	2.05 ± 0.49 ^a^	3425.75 ± 27.22 ^a^
	‘Idared’	24.2 ± 0 ^d^	n.d.	2750.9 ± 64.06 ^b^
	‘Granny Smith’	180.35 ± 14.5 ^b^	n.d.	597.25 ± 16.33 ^c^

^a^: Dry weight. Mean ± SD based on two extracts each measured twice (*n* = 4). *p* < 0.05. Different letters in each column indicate significant differences at 95% confidence level as obtained by the LSD test. n.d.—not detected.

## Data Availability

The data presented in this study are available on request from the corresponding author.

## Appendix A

**Table A1:** The *p*-values of the correlations of patulin with identified polyphenols. The *p*-values of the correlations of patulin with identified polyphenols. Statistically significant *p*-values are marked red (*p* < 0.05).

Variable	Patulin	Catechin	Epicatechin	Phloridzin	Phloretin	Procyanidine B1	Procijanidine B2	Procyanidine A2	Quercetine	Quercetin 3-β-D-glucoside	Quercetin -3-rutinoside	Myricetin	Chlorogenic acid	4-Hydroxycinnamic acid	2-6-dimethoxybenzoic acid
**Patulin**	1	0 *p =* 0.032	0 *p =* 0.000	0 *p =* 0.186	0 *p =* 0.048	0 *p =* 0.119	0 *p =* 0.003	0 *p =* 0.160	0 *p =* 0.549	0 *p =* 0.267	0 *p =* 0.025	0 *p =* 0.004	0 *p =* 0.000	0 *p =* 0.000	−1 *p =* 0.000
**Catechin**	0 *p =* 0.032	1	1 *p =* 0.000	0 *p =* 0.646	0 *p =* 0.005	0 *p =* 0.120	0 *p =* 0.002	0 *p =* 0.001	0 *p =* 0.254	0 *p =* 0.168	0 *p =* 0.094	0 *p =* 0.160	0 *p =* 0.001	1 *p =* 0.000	1 *p =* 0.000
**Epicatechin**	0 *p =* 0.000	1 *p =* 0.000	1	0 *p =* 0.648	0 *p =* 0.030	0 *p =* 0.006	1 *p =* 0.000	1 *p =* 0.000	0 *p =* 0.233	0 *p =* 0.433	0 *p =* 0.025	0 *p =* 0.000	1 *p =* 0.000	1 *p =* 0.000	1 *p =* 0.000
**Phloridzin**	0 *p =* 0.186	0 *p =* 0.646	0 *p =* 0.648	1	1 *p =* 0.000	1 *p =* 0.000	0 *p =* 0.004	0 *p =* 0.000	1 *p =* 0.000	1 *p =* 0.000	1 *p =* 0.000	0 *p =* 0.000	0 *p =* 0.009	0 *p =* 0.001	0 *p =* 0.347
**Phloretin**	0 *p =* 0.048	0 *p =* 0.005	0 *p =* 0.030	1 *p =* 0.000	1	0 *p =* 0.248	0 *p =* 0.525	0 *p =* 0.829	1 *p =* 0.000	1 *p =* 0.000	0 *p =* 0.001	0 *p =* 0.021	0 *p =* 0.543	0 *p =* 0.764	0 *p =* 0.007
**Procyanidine B1**	0 *p =* 0.119	0 *p =* 0.120	0 *p =* 0.006	1 *p =* 0.000	0 *p =* 0.248	1	1 *p =* 0.000	1 *p =* 0.000	0 *p =* 0.189	1 *p =* 0.000	1 *p =* 0.000	1 *p =* 0.000	1 *p =* 0.000	1 *p =* 0.000	0 *p =* 0.000
**Procijanidine B2**	0 *p =* 0.003	0 *p =* 0.002	1 *p =* 0.000	0 *p =* 0.004	0 *p =* 0.525	1 *p =* 0.000	1	1 *p =* 0.000	0 *p =* 0.478	0 *p =* 0.009	1 *p =* 0.000	1 *p =* 0.000	1 *p =* 0.000	1 *p =* 0.000	1 *p =* 0.000
**Procyanidine A2**	0 *p =* 0.160	0 *p =* 0.001	1 *p = *0.000	0 *p =* 0.000	0 *p =* 0.829	1 *p =* 0.000	1 *p =* 0.000	1	0 *p =* 0.123	0 *p =* 0.001	1 *p =* 0.000	1 *p =* 0.000	1 *p =* 0.000	1 *p =* 0.000	1 *p =* 0.000
**Quercetine**	0 *p =* 0.549	0 *p =* 0.245	0 *p =* 0.233	1 *p =* 0.000	1 *p =* 0.000	0 *p =* 0.189	0 *p =* 0.478	0 *p =* 0.123	1	1 *p =* 0.000	0 *p =* 0.000	0 *p =* 0.068	0 *p =* 0.998	0 *p =* 0.097	0 *p =* 0.316
**Quercetin 3-β-D-glucoside**	0 *p =* 0.267	0 *p =* 0.168	0 *p =* 0.433	1 *p =* 0.000	1 *p =* 0.000	1 *p =* 0.000	0 *p =* 0.009	0 *p =* 0.001	1 *p =* 0.000	1	1 *p =* 0.000	0 *p =* 0.001	0 *p =* 0.011	0 *p =* 0.000	0 *p =* 0.234
**Quercetin -3-rutinoside**	0 *p =* 0.025	0 *p =* 0.094	0 *p =* 0.025	1 *p =* 0.000	0 *p =* 0.001	1 *p =* 0.000	1 *p =* 0.000	1 *p =* 0.000	1 *p =* 0.000	1 *p =* 0.000	1	1 *p =* 0.000	1 *p =* 0.000	1 *p =* 0.000	0 *p =* 0.000
**Myricetin**	0 *p =* 0.004	0 *p =* 0.160	0 *p =* 0.000	0 *p =* 0.000	0 *p =* 0.021	1 *p =* 0.000	1 *p =* 0.000	1 *p =* 0.000	1 *p =* 0.068	0 *p =* 0.001	1 *p =* 0.000	1	1 *p =* 0.000	1 *p =* 0.000	1 *p =* 0.000
**Chlorogenic acid**	0 *p =* 0.000	0 *p =* 0.001	1 *p =* 0.000	0 *p =* 0.009	0 *p =* 0.543	1 *p =* 0.000	1 *p =* 0.000	1 *p =* 0.000	1 *p =* 0.998	0 *p =* 0.011	1 *p =* 0.000	1 *p =* 0.000	1	1 *p =* 0.000	1 *p =* 0.000
**4-Hydroxycinnamic acid**	0 *p =* 0.000	1 *p =* 0.000	1 *p =* 0.000	0 *p =* 0.001	0 *p =* 0.764	1 *p =* 0.000	1 *p =* 0.000	1 *p =* 0.000	1 *p =* 0.097	0 *p =* 0.000	1 *p =* 0.000	1 *p =* 0.000	1 *p =* 0.000	1	1 *p =* 0.000
**2-6-dimethoxybenzoic acid**	−1 *p =* 0.000	1 *p =* 0.000	1 *p =* 0.000	0 *p =* 0.347	0 *p =* 0.007	0 *p =* 0.000	1 *p =* 0.000	1 *p =* 0.000	1 *p =* 0.316	0 *p =* 0.234	0 *p =* 0.000	1 *p =* 0.000	1 *p =* 0.000	1 *p =* 0.000	1

## Appendix B

**Table A2:** Gradient elution conditions.

Analysis Time (min)	Solvent B Content (%)
**0**	15
**15**	35
**25**	70
**35**	15

**Table A3:** Detection wavelengths and correlation coefficients (r) calculated from the linear regression equations for the tested components.

Compound	λ	r
**IDC**	520	0.9999633
**GA**	280	0.9999866
**FA**	320	0.9999834
**CHA**	320	0.9999809
**EGC**	280	0.9991971
**CA**	320	0.9995392
**PA2**	280	0.9999338
**CAT**	280	0.9998230
**pCA**	320	0.9999413
**PHL**	280	0.9998760
**EPI**	280	0.9993037
**MY**	360	0.9997800
**RU**	360	0.9997720
**Q3G**	360	0.9999349
**PB1**	280	0.9951555
**ODC**	520	0.9995805
**QUE**	360	0.9995243
**PB2**	280	0.9997888
**P3G**	520	0.9992155

Gallic acid (GA), caffeic acid (CA), trans-ferulic acid (FA), chlorogenic acid (CHA), p-coumaric acid (pCA), Quercetin-3-rutinoside (RU), quercetin (QUE), quercetin -3-β-D-glucoside (Q3G), myricetin (MY), phloridzin (PHL), (−)-epicatechin (EPI), (+)-catechin (CAT), epigalocatechin (EGC), procyanidin B1 (PB1), procyanidin B2 (PB2), procyanidin A2 (PA2), idaein chloride (IDC), oenin chloride (ODC), pelargonidine-3-glucoside (P3G).

**Table A4:** Repeatability of solution preparation.

Compound	Extract					Average	Standard Deviation
	1	2	3	4	5		
**IDC (** **μ** **g/g)**	104.90	120.69	117.71	105.90	118.07	113.45	7.45
**GA (** **μ** **g/g)**	0.00	0.00	0.00	0.00	0.00	0.00	0.00
**FA (** **μ** **g/g)**	4.03	3.25	4.13	4.26	5.01	4.13	0.63
**CHA (** **μ** **g/g)**	516.74	518.34	542.20	534.04	540.15	530.29	12.04
**EGC (** **μ** **g/g)**	22.24	14.83	15.86	23.99	16.06	18.59	4.20
**CA (** **μ** **g/g)**	8.15	7.34	8.53	8.30	8.76	8.22	0.54
**PA2 (** **μ** **g/g)**	5.37	5.29	4.02	5.43	5.37	5.09	0.61
**CAT (** **μ** **g/g)**	46.78	47.06	45.80	44.44	48.26	46.47	1.43
**pCA (** **μ** **g/g)**	1.38	0.78	1.48	1.46	1.96	1.41	0.42
**PHL (** **μ** **g/g)**	576.94	607.06	621.91	622.19	630.21	611.66	21.13
**EPI (** **μ** **g/g)**	300.36	304.75	316.85	310.16	315.11	309.45	6.93
**MY (** **μ** **g/g)**	56.98	63.49	65.11	63.01	65.66	62.85	3.46
**RU (** **μ** **g/g)**	277.15	305.87	305.43	299.84	314.19	300.50	14.02
**Q3G (** **μ** **g/g)**	98.20	107.08	105.74	102.72	109.88	104.72	4.46
**PB1 (** **μ** **g/g)**	55.15	52.62	51.30	50.26	53.59	52.58	1.91
**ODC (** **μ** **g/g)**	1.22	1.31	1.33	1.26	1.36	1.30	0.05
**QUE (** **μ** **g/g)**	2.93	3.11	3.16	3.15	3.17	3.10	0.10
**PB2 (** **μ** **g/g)**	825.86	844.26	859.13	819.31	858.57	841.42	18.35
**P3G (** **μ** **g/g)**	0.00	0.00	0.00	0.00	0.00	0.00	0.00

Gallic acid (GA), caffeic acid (CA), trans-ferulic acid (FA), chlorogenic acid (CHA), p-coumaric acid (pCA), Quercetin-3-rutinoside (RU), quercetin (QUE), quercetin -3-β-D-glucoside (Q3G), myricetin (MY), phloridzin (PHL), (−)-epicatechin (EPI), (+)-catechin (CAT), epigalocatechin (EGC), procyanidin B1 (PB1), procyanidin B2 (PB2), procyanidin A2 (PA2), idaein chloride (IDC), oenin chloride (ODC), pelargonidine-3-glucoside (P3G).

**Table A5:** Measurement repeatability.

Compound	Injection				Average	Standard Deviation
	1	2	3	4		
**IDC (μg/g)**	104.90	104.59	103.96	102.37	103.96	1.13
**GA (μg/g)**	0.00	0.18	0.16	0.27	0.15	0.11
**FA (μg/g)**	4.03	3.16	4.26	3.09	3.63	0.60
**CHA (μg/g)**	516.74	517.12	517.86	513.99	516.43	1.69
**EGC (μg/g)**	22.24	22.19	24.06	14.83	20.83	4.09
**CA (μg/g)**	8.15	7.29	8.32	7.32	7.77	0.54
**PA2 (μg/g)**	5.37	7.04	6.26	5.53	6.05	0.76
**CAT (μg/g)**	46.78	48.13	47.54	44.48	46.73	1.60
**pCA (μg/g)**	1.38	0.72	1.47	0.73	1.08	0.40
**PHL (μg/g)**	576.94	580.47	578.12	576.40	577.98	1.81
**EPI (μg/g)**	300.36	301.76	298.77	296.40	299.32	2.30
**MY (μg/g)**	56.98	57.21	58.89	53.31	56.60	2.35
**RU (μg/g)**	277.15	280.05	279.33	277.97	278.62	1.31
**Q3G (μg/g)**	98.20	97.46	97.66	98.26	97.89	0.40
**PB1 (μg/g)**	55.15	54.30	55.13	50.72	53.83	2.11
**ODC (μg/g)**	1.22	1.22	1.13	1.19	1.19	0.04
**QUE (μg/g)**	2.93	3.10	3.04	2.96	3.01	0.08
**PB2 (μg/g)**	825.86	824.54	818.20	799.24	816.96	12.28
**P3G (μg/g)**	0.00	0.00	0.00	0.00	0.00	0.00

Gallic acid (GA), caffeic acid (CA), trans-ferulic acid (FA), chlorogenic acid (CHA), p-coumaric acid (pCA), Quercetin-3-rutinoside (RU), quercetin (QUE), quercetin -3-β-D-glucoside (Q3G), myricetin (MY), phloridzin (PHL), (−)-epicatechin (EPI), (+)-catechin (CAT), epigalocatechin (EGC), procyanidin B1 (PB1), procyanidin B2 (PB2), procyanidin A2 (PA2), idaein chloride (IDC), oenin chloride (ODC), pelargonidine-3-glucoside (P3G).

**Table A6:** Accuracy and the trueness of HPLC method for the detection of apple polyphenols.

Compound	Measured Value (μg/mL)	Expected Value (μg/mL)	Exploitation (%)
**IDC**	11.83	12.85	92.0
**GA**	10.52	10.62	99.0
**FA**	10.76	11.06	97.3
**CHA**	65.26	64.11	101.8
**EGC**	15.86	15.28	103.8
**CA**	11.49	11.35	101.2
**PA2**	15.34	15.28	100.4
**CAT**	15.55	16.15	96.3
**pCA**	11.48	10.66	107.7
**PHL**	77.69	74.18	104.7
**EPI**	46.56	47.40	98.2
**MY**	16.76	16.78	99.9
**RU**	40.90	40.35	101.4
**Q3G**	20.09	20.19	99.5
**PB1**	15.61	15.49	100.7
**ODC**	8.74	9.77	89.5
**QUE**	11.46	10.49	109.2
**PB2**	95.40	96.59	98.8
**P3G**	9.59	10.18	94.2

Gallic acid (GA), caffeic acid (CA), trans-ferulic acid (FA), chlorogenic acid (CHA), p-coumaric acid (pCA), Quercetin-3-rutinoside (RU), quercetin (QUE), quercetin -3-β-D-glucoside (Q3G), myricetin (MY), phloridzin (PHL), (−)-epicatechin (EPI), (+)-catechin (CAT), epigalocatechin (EGC), procyanidin B1 (PB1), procyanidin B2 (PB2), procyanidin A2 (PA2), idaein chloride (IDC), oenin chloride (ODC), pelargonidine-3-glucoside (P3G).
